# Development and Validation of a Nomogram Model Based on Hematological Indicators for Predicting the Prognosis of Diffused Gliomas

**DOI:** 10.3389/fsurg.2022.803237

**Published:** 2022-04-13

**Authors:** Song Han, Fang-wen Qu, Peng-fei Wang, Ying-xin Liu, Shou-wei Li, Chang-xiang Yan

**Affiliations:** ^1^Department of Neurosurgery, Sanbo Brain Hospital, Capital Medical University, Beijing, China; ^2^Grade 2018, Medical College, Qingdao University, Qingdao, China

**Keywords:** nomogram, prognosis, hematological, predictive modeling, gliomas

## Abstract

**Background:**

Diffused gliomas are aggressive malignant brain tumors. Various hematological factors have been proven to predict the prognosis of patients with gliomas. The aim of this study is to integrate these hematological markers and develop a comprehensive system for predicting the prognosis of patients with gliomas.

**Method:**

This retrospective study included 723 patients pathologically diagnosed with diffused gliomas. Hematological indicators were collected preoperatively, including neutrophil-to-lymphocyte ratio (NLR), lymphocyte-monocyte ratio (LMR), platelet-to-lymphocyte ratio (PLR), albumin globulin ratio (AGR), platelet distribution width (PDW), red blood cell distribution width (RDW), fibrinogen (FIB), and prognostic nutritional index (PNI). Least absolute shrinkage and selection operator (LASSO) Cox was applied to screen the hematological indicators for a better prediction of patients' prognosis and to build an inflammation-nutrition score. A nomogram model was developed to predict the overall survival (OS), which included age, tumor grade, IDH-1 mutations, and inflammation-nutrition score.

**Result:**

Patients were randomly divided into a primary cohort (*n* = 509) and a validation cohort (*n* = 214). There was no difference in age and IDH-1 mutation frequency between the cohorts. In the primary cohort, NLR, LMR, AGR, FIB, and PNI were selected to build an inflammation nutrition score. Patients with a high-risk inflammation-nutrition score had a short median OS of 17.40 months compared with 27.43 months in the low-risk group [*HR* 2.54; 95% *CI* (1.91–3.37); *p* < 0.001]. Moreover, age, tumor grade, IDH-1 mutations, and inflammation-nutrition score were independent prognostic factors in the multivariate analysis and thus were included in the nomogram model. The nomogram model showed a high prediction value with a Harrell's concordance index (C-index) of 0.75 [95% *CI* (0.72–0.77)]. The validation cohort supported these results.

**Conclusion:**

The prognostic nomogram model provided a high prognostic predictive power for patients with gliomas.

## Introduction

Diffused gliomas, the most common type of primary brain tumors, have a poor prognosis (1). Glioblastomas represent almost half of the newly diagnosed cases of gliomas, and are the most malignant type of brain cancer (2). Effective prediction of prognosis helps in facilitating the treatment, enhancing the prognosis, and prolonging the survival.

There is increasing evidence that preoperative hematological parameters can be used to predict the prognosis of gliomas. The neutrophil-to-lymphocyte ratio (NLR), platelet-to-lymphocyte ratio (PLR), red blood cell distribution width (RDW), platelet distribution width (PDW), and fibrinogen (FIB) (3–7) had negative predictive values, but prognostic nutritional index (PNI), lymphocyte-monocyte ratio (LMR), and albumin globulin ratio (AGR) had positive predictive values (3, 8–10). Among these indicators, NLR has a role in inflammation and affects the tumor microenvironment, while LMR is associated with immunosurveillance. However, some of these parameters did not have proven prognostic value in previous studies (11–13), which suggests a weak prognostic value for single parameters. Accordingly, multiple indicators have been used simultaneously to provide an accurate and stable prognostic model. A developed nomogram predictive model combining albumin and LMR could effectively predict survival in patients with breast cancer (14). However, few studies have included more than two prognostic factors for the prediction of prognosis in patients with gliomas.

In this study, we aimed to develop a predictive model by integrating eight hematological scores to enhance the prediction of prognosis in patients with diffused gliomas.

## Materials and Methods

### Patient Selection

This study retrospectively reviewed 723 patients who were admitted to the Sanbo Brain Hospital, Capital Medical University. All the patients were pathologically diagnosed with gliomas. The inclusion criteria were: (1) patients who had completed routine blood tests, blood biochemical tests, and coagulation tests before surgery; and (2) those who underwent surgery. The exclusion criteria were patients with co-existing diseases, including hematological disorders, autoimmune diseases, infection, and renal or hepatic dysfunction. Baseline clinicopathologic data were obtained from medical records, including age, sex, tumor grade, and IDH-1 mutation status, which was evaluated by immunohistochemistry staining (15). The histopathological diagnosis of gliomas was based on the World Health Organization (WHO) classification (16).

The study was approved by the ethics committee of Sanbo Brain Hospital, Capital Medical University (approval no. SBNK-2018-003-01). The study was conducted in adherence to the guidelines set forth by the Declaration of Helsinki. Written informed consent was obtained from all the participants.

### Data Collection

Hematological markers were collected preoperatively. The markers were calculated as follows: NLR was calculated as neutrophil count/lymphocyte count, PLR was calculated as platelet count/lymphocyte count, LMR was calculated as lymphocyte count/monocyte count, AGR was calculated as albumin/globulin, and PNI was calculated as 10 × serum albumin value (g/dl) + 0.005 × peripheral lymphocyte count (per mm^3^).

### The Construction of Inflammation-Nutrition Score

The LASSO regression model was used to screen the most significant prognostic factors in the primary group, such as NLR, LMR, AGR, FIB, and PNI. The coefficients were obtained with a minimum lambda value of 0.02134553 (**Figures 2A,B**). A formula was derived based on those five parameters to calculate the inflammation-nutrition score, as follows: (0.002410232 × NLR) + (-0.02908379 × LMR) + (-0.16049733 × AGR) + (0.25193846 × FIB (G/I)) + (-0.03974138 × PNI).

### Statistical Analysis

All data analyses were performed using R-language (version 4.0, Boston, MA). Unpaired Student's *t*-tests and variance analysis were used to analyze differences in continuous variables between cohorts, while the chi-square test was used to analyze discrete variables. The optimal cut-off value for predicting survival of the inflammation-nutrition score was determined using X-tile software (version 3.4.1, Yale University, New Haven, Connecticut). LASSO COX analysis and the nomogram were performed using the glmnet and rms packages (version 3.2.1) in R software. The Kaplan–Meier method was used to calculate the overall survival (OS). Statistical significance was set at *p* < 0.05.

## Results

### Patient Characteristic

**Figure 1:**
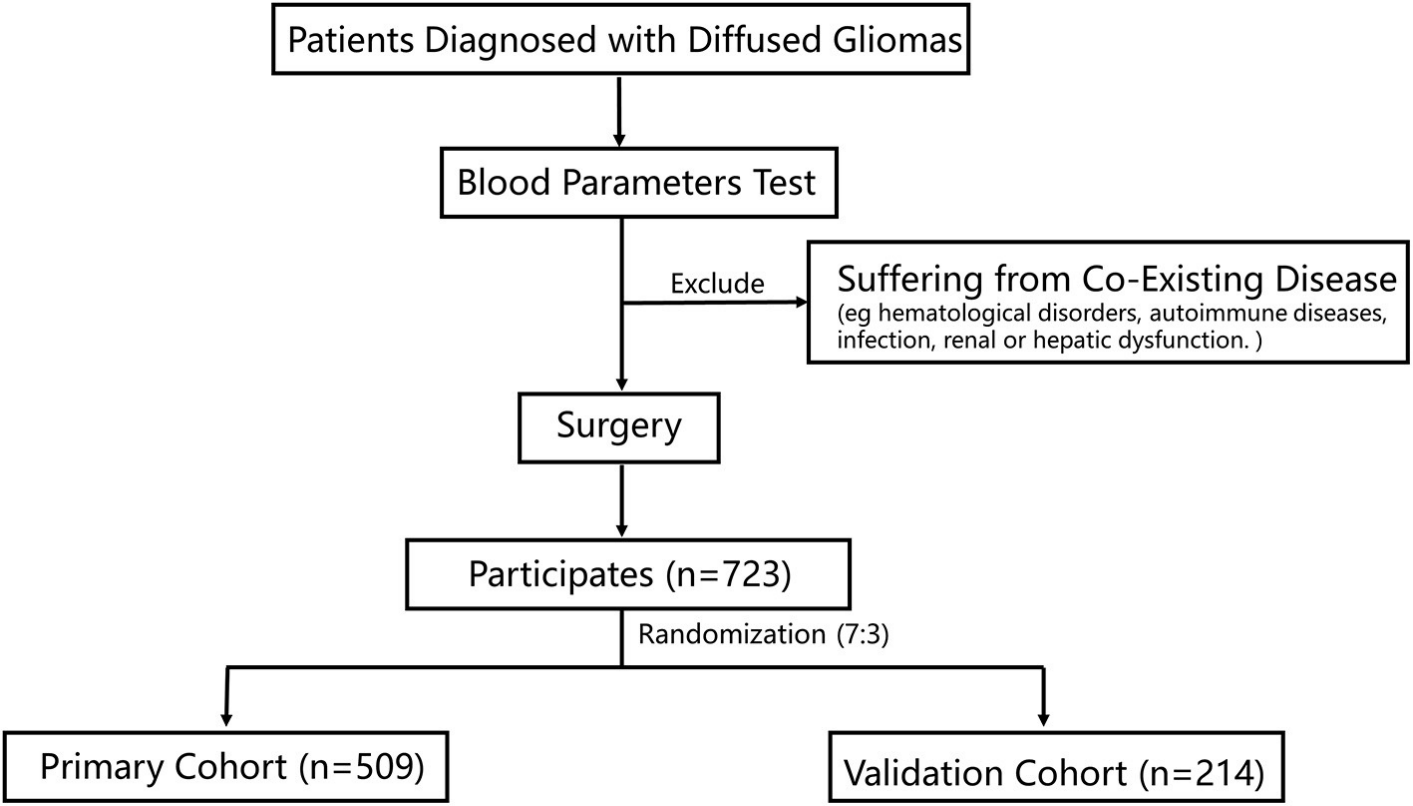
A flow chart of the process of patient selection.

A total of 723 patients were included in this study (Figure 1). Of these, 308 patients (42.6%) were women, and the mean age of all participants was 40.0 years. The participants were randomly divided into the primary cohort (*n* = 509) and the validation cohort (*n* = 214). The characteristics of the patients were comparable between the primary and validation cohorts (Table 1). The median survival time was 19.50 months (9.03–50.60) in the primary cohort and 17.13 months (10.03–44.83) in the validation cohort.

**Table 1:** Baseline characteristics.

	**Primary cohort (*n* = 509)**	**Validation cohort (*n* = 214)**	***p*-value**
Age	45.94 ± 13.08	45.44 ± 13.85	0.711
Women (%)	218 (42.8%)	90 (42.1%)	0.869
NLR	2.54 ± 1.97	2.34 ± 1.26	0.137
PLR	134.37 ± 66.18	132.20 ± 57.86	0.677
LMR	5.20 ± 2.54	5.06 ± 2.31	0.533
RDW	13.85 ± 1.42	13.78 ± 1.25	0.927
PDW	15.98 ± 1.17	15.92 ± 1.24	0.066
PNI	52.07 ± 5.36	51.87 ± 5.47	0.513
AGR	1.83 ± 0.35	1.84 ± 0.37	0.560
FIB (g/L)	2.46 ± 0.65	2.44 ± 0.64	0.800
IDH-1 mutation	209 (41.1%)	84 (39.3%)	0.679
Overall survival (Median, months)	19.50 (9.03 – 50.60)	17.13 (10.03 – 44.83)	0.120

### Survival Analysis of the Inflammation-Nutrition Score

**Figure 2:**
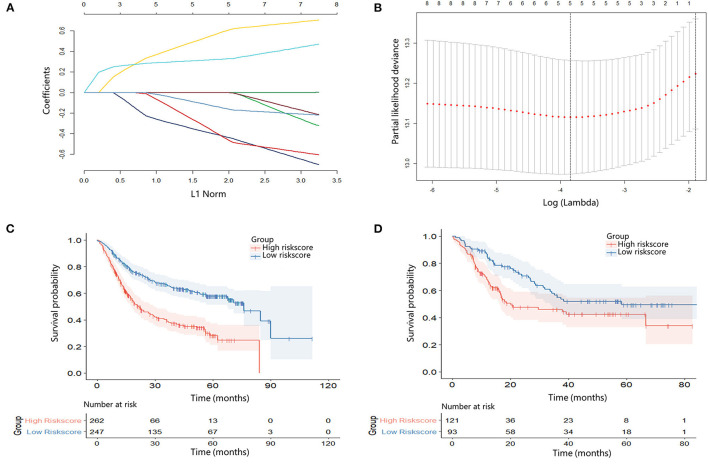
**(A)** Least absolute shrinkage and selection operator (LASSO) coefficient profiles of eight hematological indicators. **(B)** 10-fold cross-validation for tuning parameter selection in the LASSO model. **(C)** Kaplan–Meier survival curve according to the inflammation-nutrition score in the primary cohort. High inflammation-nutrition score, *n* = 262; low inflammation-nutrition score, *n* = 247. **(D)** Kaplan–Meier survival curve according to the inflammation-nutrition score in the validation cohort. High inflammation-nutrition score, *n* = 121; low inflammation-nutrition score, *n* = 93.

The inflammation-nutrition score was divided into two groups based on the optimum cut-off value, which was calculated using X-tile software (primary, −1.58; validation, −1.92). In the primary group, the median OS in the high-score group was 17.40 months (7.13–18.37) compared with 27.43 months (12.50–56.30) in the low-score group. The results indicated that patients with a low-risk score had a better prognosis [*HR* 2.54; 95% *CI* (1.91–3.37); *p* < 0.001]. In the validation group, the median OS was estimated to be 13.80 months (8.00–36.17) for the high-score group and 28.70 months (14.50–58.00) for the low-score group. The results were in accordance with the primary group [*HR* 1.74; 95% *CI* (1.15–2.62); *p* = 0.009] (Figures 2C,D).

### Development of Nomogram for OS

**Figure 3:**
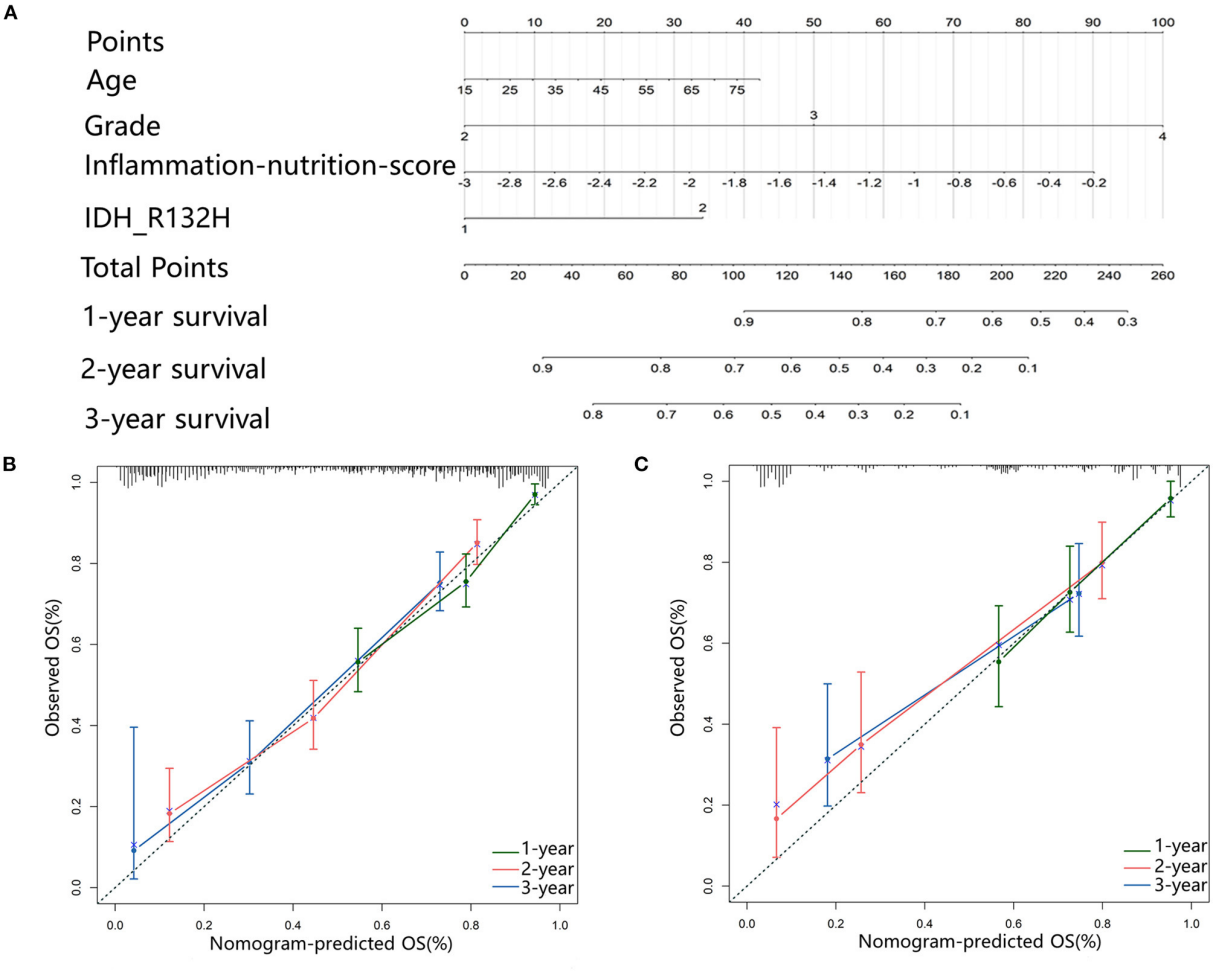
**(A)** Nomogram for predicting the 1-, 3-, and 5-year prognosis for patients with Gliomas. **(B,C)** Calibration plots represent the consistency between the predicted results and the observations calibrated for each model. They showed the accuracy of nomogram regarding to 1-, 3-, and 5-year OS both in primary **(B)** and validation **(C)** cohort. Dashed line at 45° represents perfect prediction, and the actual performances of our nomogram are red, blue, and green lines.

In the multivariate analysis, age, tumor grade, IDH-1 mutations, and inflammation-nutrition score were found to be independently associated with the OS (Table 2, Supplementary Figure 1). A nomogram was constructed based on age, tumor grade, IDH-1 mutations, and inflammation-nutrition scores (Figure 3A). In the nomogram, the score range for the inflammation-nutrition score was between 0 and 90.

**Table 2:** Univariate and multivariate cow analysis of primary cohort.

	**Univariate analysis**		**Multivariate analysis**	
	**HR (95% CI)**	***p*-value**	**HR (95% CI)**	***p*-value**
Age^*^	1.038 (1.027 – 1.358)	<0.001	1.011 (1.000 – 1.022)	0.043
**Gender**				
Female	0.977 (0.789 – 1.120)	0.832		
Male	Reference			
**Grade**				
GBM	2.955 (2.442 – 3.576)	<0.001	2.345 (1.914 – 2.873)	<0.001
LGG	Reference			
**IDH-1 R132H**				
Wild-type	3.000 (2.258 – 3.987)	<0.001	1.789 (1.318 – 2.429)	<0.001
Mutation	Reference			
Inflammation nutrition score^*^	2.538 (1.910 – 3.366)	<0.001	1.731 (1.188 – 2.523)	0.004

* *Markers were regarded as continuous variable*.

The prognostic value of the nomogram was assessed using the Harrell's concordance index (C-index) of 0.75. The calibration plot of the nomogram showed that the estimated 1-, 3-, and 5-year OS were highly consistent with the ideal OS curve and the calibration plot showed an agreement between the prediction and the observation (Figures 3B,C).

## Discussion

Despite the great advances in the diagnosis and treatment of gliomas, there is still a lack of a comprehensive scoring system that can accurately predict the prognosis of patients. In this study, the inflammation-nutrition score which was developed to predict OS in patients with gliomas, showed an independent prognostic value. We combined preoperative NLR, FIB, AGR, LMR, and PNI to build a novel score. To increase the accuracy of the predicted prognosis, we integrated the score with several clinicopathological variables to develop a nomogram model. The model showed a high prediction accuracy in the validation cohort.

A number of nomogram models based on hematological indicators have been recently established to predict the prognosis of patients with malignant tumors. After establishing the nomogram, the C-index was calculated for verification. For example, a predictive score combining neutrophils, lymphocytes, platelets, and fibrinogen for gastric cancer had a C-index of 0.72 (17), and a scoring system based on lymphocyte, neutrophil, and platelet counts for cervical cancer had a C-index of 0.64 (18). Our study combined more hematological parameters based on results of previous studies. The C-index in our study was 0.75, indicating that a more comprehensive combination of hematological parameters has a higher predictive ability.

The underlying mechanism for the prognostic value of the scoring system remains unclear. Neutrophils and monocytes have been shown to be immune suppressors in the tumor microenvironment (19, 20). Tumor-infiltrating lymphocytes cause cytotoxic cell death, resulting in a significant immune response (21). Albumin concentration is a sensitive marker for the assessment of patients' nutritional status. In patients with esophageal cancer, high albumin levels have higher total protein levels and a higher quality of life than those with low albumin levels (22). PNI is a nutritional indicator that positively correlates with albumin levels and is viewed as a novel independent prognostic factor for predicting OS in patients with malignant melanoma (23). Studies have shown that high plasma fibrinogen levels can suppress anti-tumor immunity and facilitate tumor progression and metastasis (24, 25).

There are certain limitations to this study. First, it was a retrospective, single-center study, which suggests a potential recall bias. However, the validation is an efficient measure to address this limitation. Further prospective, multicenter studies are needed to confirm these results. Second, the relationship between the inflammation-nutrition score and other hematology-based scores was not investigated. Third, there was no fixed cutoff value of inflammation-nutrition score, which made it difficult into practice usage.

In conclusion, the inflammation-nutrition score is a simple, independent, and non-invasive prognostic scoring system for patients with gliomas. The nomogram model further improved the prediction of prognosis for patients with gliomas.

## Data Availability Statement

The original contributions presented in the study are included in the article/Supplementary Materials, further inquiries can be directed to the corresponding author.

## Ethics Statement

The studies involving human participants were reviewed and approved by Ethics Committee of Sanbo Hospital of Capital Medical University (No. SBNK-2018-003-01). The patients/participants provided their written informed consent to participate in this study. Written informed consent was obtained from the individual(s) for the publication of any potentially identifiable images or data included in this article.

## Author Contributions

SH and C-xY: conception and design. P-fW and F-wQ: collection and acquisition of data. Y-xL and S-wL: data analysis and interpretation. All authors: drafting and approval of the manuscript.

## Funding

This work was supported by grants from the National Key Technology Research and Development Program of the Ministry of Science and Technology of China (No. 2014BAI04B01).

## Conflict of Interest

The authors declare that the research was conducted in the absence of any commercial or financial relationships that could be construed as a potential conflict of interest.

## Publisher's Note

All claims expressed in this article are solely those of the authors and do not necessarily represent those of their affiliated organizations, or those of the publisher, the editors and the reviewers. Any product that may be evaluated in this article, or claim that may be made by its manufacturer, is not guaranteed or endorsed by the publisher.

## Supplementary Material

Supplementary Figure 1 Forest plot of multivariate COX analysis.
