# Supplementary figures and images for: Development and Validation of a Nomogram Model Based on Hematological Indicators for Predicting the Prognosis of Diffused Gliomas
Source: Front Surg. 2022 Apr 13;9:803237. doi: 10.3389/fsurg.2022.803237 (PMC9043458; doi:10.3389/fsurg.2022.803237)

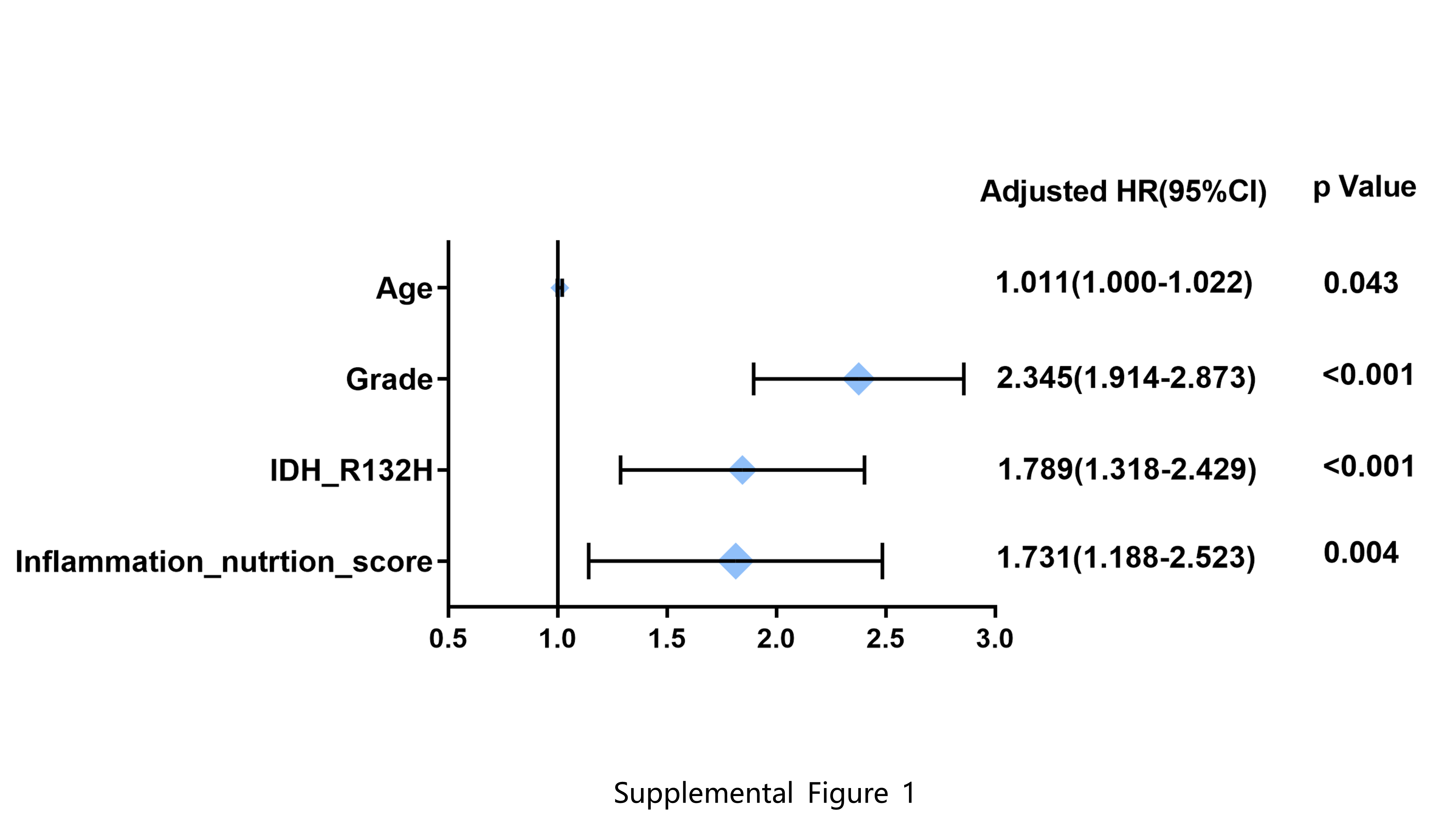

Supplement: Supplementary Figure 1 — Forest plot of multivariate COX analysis. [file Image_1.TIF]
